# Triplet RNA Lipid Nanoparticles for Locoregional Cancer Immunotherapy

**DOI:** 10.1002/smsc.202500506

**Published:** 2025-12-12

**Authors:** Adam A. Walters, Yue Qin, Amer F. Saleh, Calvin C. L. Cheung, Qingyang Lyu, Ziyi Zhu, Hiba A. M. Gafar, Julie Tzu‐Wen Wang, Khuloud T. Al‐Jamal

**Affiliations:** ^1^ Institute of Pharmaceutical Science Faculty of Life Sciences & Medicine King's College London Franklin‐Wilkins Building, 150 Stamford Street London SE1 9NH UK; ^2^ Cell Therapy Safety Clinical Pharmacology and Safety Sciences BioPharmaceuticals R&D AstraZeneca Cambridge CB2 0AA UK; ^3^ JC STEM Lab of Nanomedicine for Advanced Therapy Department of Pharmacology and Pharmacy LKS Faculty of Medicine The University of Hong Kong Hong Kong SAR China

**Keywords:** immunotherapy, intratumoral, lipid nanoparticles, poly (I:C)

## Abstract

Ionizable lipid nanoparticles (LNPs) are a proven means of delivering nucleic acid‐based therapeutics. This project aims to expand the LNP platform for the delivery of immunostimulatory polyinosinic‐polycytidylic acid (pIpC). It is demonstrated that pIpC could be successfully incorporated into LNPs with minimal modification to existing protocols. LNPs encapsulating pIpC (pIpC‐LNPs) exhibit a spherical shape with a diameter under 200 nm. When administered intratumorally, pIpC‐LNPs are significantly more potent than the soluble adjuvant, resulting in complete remission in 25% of tumors. To identify potential synergistic targets, T cell activation markers are screened following pIpC‐LNP treatment. OX40 and CD27 are strongly upregulated and associated with intratumoral pIpC‐LNP administration. Furthermore, direct treatment of a cancer cell line with pIpC‐LNPs results in upregulation of the immunosuppressive PDL1. To develop a comprehensive RNA‐based immunotherapeutic strategy, LNPs are formulated with mRNAs encoding CD70 (the CD27 ligand) and OX40L, or with siRNA targeting PDL1, and are evaluated in combination. Tumor growth reduction is observed when pIpC‐LNPs are combined with siPDL1. This study demonstrates the potential of a triplet RNA platform‐comprising immunostimulatory RNA, mRNA, and siRNA, delivered via a single versatile LNP. The data support development of pIpC‐LNPs as potent intratumoral therapeutics and highlight several potential synergistic targets.

## Graphical abstract

LNPs deliver the immunoadjuvant pIpC into tumors to activate local cells. After activation, additional RNA (siRNA or mRNA) can modulate immune pathways by suppressing immunoinhibitory targets or inducing immunostimulatory ones. This coordinated “triplet RNA” approach may enhance immune activation within the tumor microenvironment.

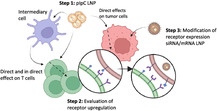

## 1 Introduction

The use of nucleic acids which can potently activate the immune system represents an untapped resource in cancer immunotherapy. Immunostimulatory nucleic acids fall into a range of categories including agonists of the toll‐like receptors (TLR), RIG‐like receptors (RLR), and stimulator of interferon genes (STING). The nucleic acid‐specific TLRs are predominantly expressed in the endosome of subsets of immune cells, and they include TLR3, 7/8, and 9, recognizing dsRNA, ssRNA, and unmethylated DNA (CpG), respectively. In contrast, RLR, such as RIG‐I and MDA‐5, have a broader cellular expression and are localized to the cytosol, both receptors recognize RNA of differing physical and chemical parameters, including length and terminal phosphorylation. Double‐stranded RNA is a highly potent immunostimulatory molecule capable of being detected by TLR3 and cytosolic RLRs, such as MDA‐5.^[^ ^1^ ^]^ After recognizing dsRNA, TLR3 recruits TIR‐domain‐containing adapter inducing interferon‐β (TRIF) and activates the transcription factors interferon regulatory factor 3 (IRF‐3), NF‐κB, and activator protein 1 (AP‐1), leading to the induction of type I interferon (IFN) and proinflammatory cytokines.^[^ ^1^, ^2^ ^]^ In the cytosol, dsRNA is sensed by MDA‐5 which engages the adapter protein mitochondrial antiviral‐signaling (MAVS). This initiates a signal cascade that activates transcription factors, including IRF‐3 and NF‐κB, ultimately leading to the induction of type I IFN.^[^ ^3^ ^]^

The synthetic analogue of dsRNA is double‐stranded polyinosinic acid (pI) and poly cytidylic acid (pC, pIpC).^[^ ^4^ ^]^ Due to its potency, pIpC holds great promise in cancer immunotherapy; however, to obtain a safe and effective pIpC formulation, there are several important factors that need to be considered. First, as pIpC can be hydrolyzed by nuclease in serum, it is essential to protect pIpC from physiological fluids while not impeding its efficacy. Secondly, systemic administration of free pIpC is associated with excessive immune stimulation and dose‐dependent toxicity. Finally, to reach its full potential, pIpC must be delivered to both cytosolic and endosomal receptors. Various types of vectors have been developed to satisfy some or all of these criteria. The most commonly utilized formulation is pIpC combined with poly‐l‐lysine and carboxymethylcellulose (poly ICLC, Hiltonol) which has been used in several clinical trials, including vaccination and intratumoral therapy.^[^ ^5^, ^6^ ^]^ pIpC has also been formulated with traditional cationic transfection reagents, such as polyethyleneimine PEI.^[^ ^7^ ^]^ Notably, the PEI/pIpC formulation, BO‐112, has successfully reached clinical trials.^[^ ^8^ ^]^ BO‐112 has been demonstrated to be highly effective when delivered intratumorally and has been shown to be more potent than soluble pIpC in a number of murine models.^[^ ^9^ ^]^ Furthermore, local treatment of tumor with BO‐112 can improve the resolution of distal tumors, an effect which is enhanced by coadministration of immune checkpoint targeting monoclonal antibodies (mAbs).^[^ ^9^, ^10^ ^]^

Delivery of immunostimulatory nucleic acid intratumorally overcomes systemic immune activation and related adverse events while also achieving high local concentrations which would otherwise be impossible with systemic administration. In addition to pIpC, a TLR9 agonist (SD‐101) has been trialled clinically using local delivery with minimal toxicity observed.^[^ ^11^ ^]^ It has been shown that intratumoral delivery of CpG causes the upregulation of immune checkpoint OX40 on the T cell surface and that the potency of CpG can be augmented through simultaneous delivery of anti‐OX40 mAb.^[^ ^12^, ^13^ ^]^ It is unknown whether this is specific to CpG or a more general response to TLR/inflammatory signaling. Furthermore, it is unclear whether there are other immune checkpoints which can be targeted following the administration of immunostimulatory nucleic acid.

Ionizable lipid nanoparticles (LNP) are the most clinically advanced platform for nucleic acid delivery, capable of fulfilling all requirements outlined above; most notably, they are effective at delivering nucleic acid to the cytosol, as demonstrated by the numerous mRNA/siRNA applications.^[^ ^14^, ^15^, ^16^ ^]^ LNPs have previously been used to deliver CpG in prophylactic vaccine models, where it was shown that LNP formulation could significantly increase the potency of the agonist.^[^ ^17^ ^]^ However, as CpG is primarily detected by TLR9 in the endosome, the LNP platform may be more suited to the delivery of nucleic acid which can bind endosomal and cytosolic receptors such as pIpC. In addition to delivering immunostimulatory nucleic acid, it has been demonstrated that LNPs can replace mAbs in immune checkpoint blockade by either expressing costimulatory molecules or knocking down coinhibitory molecules to result in synergistic outcomes.^[^ ^18^, ^19^, ^20^ ^]^ Therefore, immunostimulatory nucleic acids and nucleic acid‐based immune checkpoint blockade can be delivered using a single platform. Such an approach will greatly streamline the clinical development of this strategy.

This study aims to develop a pIpC delivery platform based on clinically acceptable ionizable lipid nanoparticles and to then coadminister it with LNPs delivering nucleic acid‐based immune checkpoint blockade. We hypothesize that cytosolic delivery of pIpC will enhance potency due to engagement of cytosolic dsRNA receptors and that combination with immune checkpoint targeting nucleic acid will improve local cancer therapy. We first formulated pIpC in LNPs and tested it in vivo in comparison with a soluble agonist. We then identified immune checkpoint targets which are upregulated in response to pIpC and developed a nucleic acid‐based immune checkpoint targeting LNP. The LNP combinations were tested in vitro and finally in an in vivo cancer model.

## 2 Results and Discussion

### 2.1 Immunoadjuvant pIpC Significantly Reduces Tumor Growth When Administered as LNPs

**Scheme 1:**
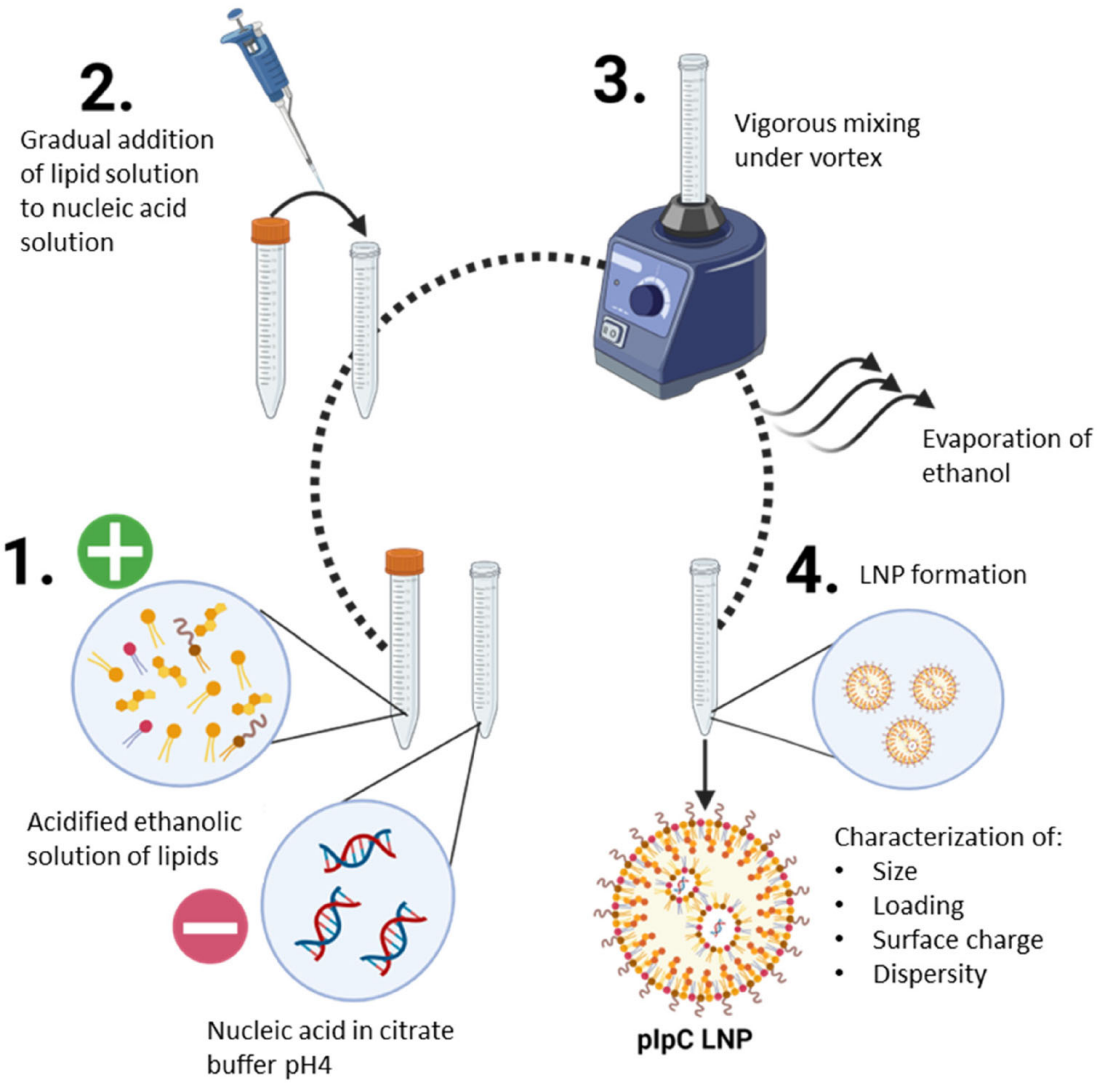
Preparation of pIpC‐LNP. An acidified solution of lipids (Dlin‐MC3‐DMA, DOPE, Cer16‐PEG2000, Cholesterol) in ethanol was prepared. Ionizable lipids become positively charged at low pH. Nucleic acid was diluted in citrate buffer pH4; nucleic acid is inherently negatively charged due to the presence of phosphate groups (1). The lipid solution is gradually added to the nucleic acid solution followed by vigorous mixing with vortex (2, 3). Following the final addition of lipid solution, the ethanol is removed by evaporation under nitrogen leading to the formation of pIpC‐LNP (4). Particles were finally characterized.

**Figure 1:**
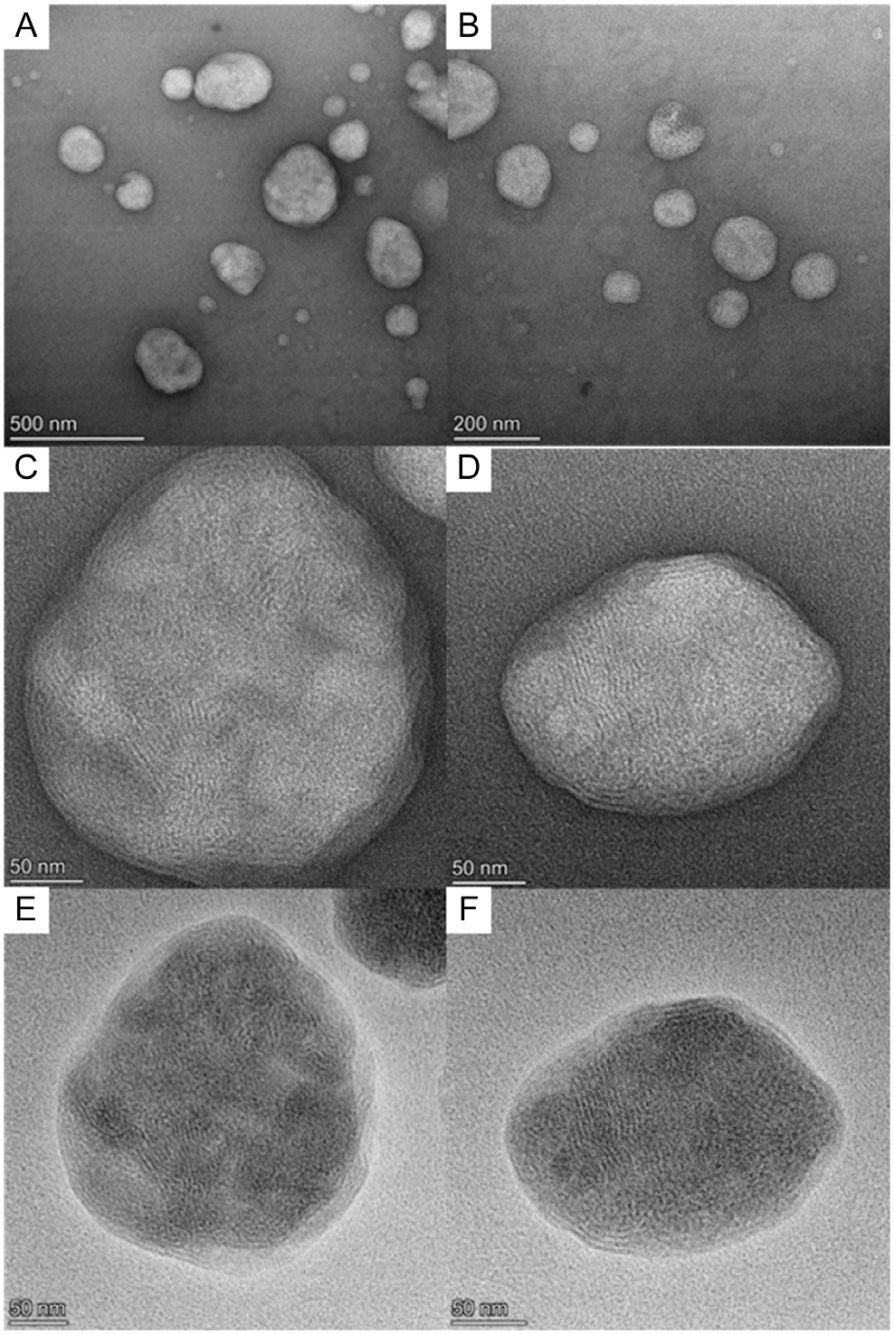
TEM images of freshly prepared pIpC‐LNP negative‐stained with PTA. A,B) Micrographs showing group of pIpC‐LNPs. C,D) High magnification images of selected pIpC‐LNP. Concentric patterns at the surface and lattice fringe (parallel lines) at the core can be observed. E,F) Inverted grayscale images of (C,D) as a visual aid.

Utilizing the LNP formulation and methods we described previously (**Scheme** **1**) for mRNA/siRNA delivery, we first assessed whether double‐stranded RNA analogue pIpC could be incorporated into LNPs. We selected to utilize an LNP formulation comprising Dlin‐MC3‐DMA, cholesterol, and C16‐ceramide at a molar ratio of 35:46.5:16:2.5, in accordance with our previous work.^[^ ^21^ ^]^ As shown in Table S1, Supporting Information, pIpC could be incorporated with a 78.1 ± 3.8% encapsulation efficiency yielding LNPs of an average size of 120.5 ± 0.4 d.nm with a surface charge of −7.17 ± 4.8 at physiological pH. The LNPs had a low PDI (<0.1) indicating a uniform nanoparticle suspension had been obtained. LNPs for ssRNA (mRNA) and siRNA, used as controls, have been previously formulated using this method and are described elsewhere. LNPs encapsulating pIpC (pIpC‐LNP) were visualized by transmission electron microscopy (TEM) with phosphotungstic acid negative staining (**Figure** **1**). Spherical particles of 80–200 nm were clearly observed (Figure 1A,B). At higher magnification (Figure 1C,D) of individual particles, concentric patterns at the surface and lattice fringes (regularly spaced parallel lines) at the core were observed. Additional grayscale inverted images on the same particles were acquired as a visual aid (Figure 1E,F). The images suggested the pIpC‐LNPs exhibited a mixed lamellar and inverted hexagonal phases.

Neither the lipid components nor the method needed to be altered to accommodate this switch. This was expected as pIpC is composed of RNA analogues and maintains the negative charge essential for LNP formulation. However, it should be noted that the formulation was not optimized for pIpC delivery and greater immunostimulation may be achieved using a bespoke formulation designed through a systematic design‐of‐experiments approach as has been performed for mRNA and siRNA.^[^ ^22^, ^23^ ^]^ Indeed, the optimization of LNP formulation to maximize immunostimulation is not widely performed as, in most cases, the objective is to reduce innate immune activation which may impede expression of mRNA or result in adverse inflammation.^[^ ^24^ ^]^ Improved responses may be observed when next‐generation ionizable lipids such as SM‐102 or ALC‐315 are used; these have been shown to be superior to MC3 in terms of protein expression.^[^ ^25^ ^]^ The LNPs developed in this study were comparable in terms of physical parameters to those previously described, suggesting either the lipids or the process is the key determinant of the LNP characteristics, rather than the payload.

**Figure 2:**
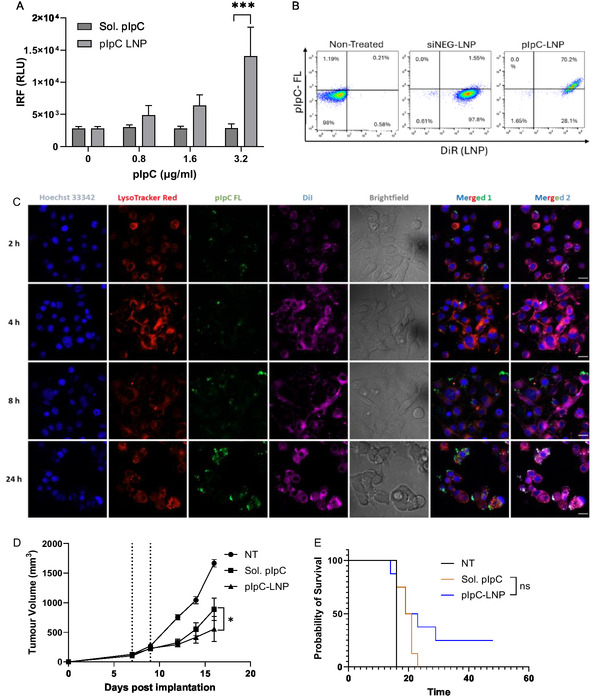
Nanoparticulate pIpC induces IRF responses in vitro and significantly reduces tumor growth resulting in increased survival after IT injection. A) THP‐1 dual monocytic cells were simulated with either soluble pIpC (Sol. pIpC) or pIpC incorporated into a lipid nanoparticle (pIpC‐LNP) at a range of concentrations for 24 h. Following the simulation, supernatant was removed and assayed for IRF reporter using Quanti‐Luc reagent. Data plotted represents the relative light unit (RLU) readout from *n* = 3 repeat studies. Data was analyzed with a 2‐way ANOVA followed by Sidak's multi comparison test. LNPs were formulated with either negative siRNA (siNEG) or pIpC conjugated to fluorescein (pIpC‐FL); DiR dye was incorporated at a final concentration of 1% molar ratio. B16F10 cells were incubated with LNP (1 μg mL^−1^) for 24 h before being stained with Zombie Aqua viability dye; LNP uptake was assessed using flow cytometry. B) The uptake of LNP in viable cells. C) Confocal images of B16‐F10 cells incubated with DiI‐labeled pIpC‐FL‐LNP for 2, 4, 8, and 24 h. The cell nuclei were stained using Hoechst 33342 (Blue); the endo/lysosomes were stained using LysoTracker Red (red); pIpC‐FL and LNP were tracked using lipophilic dye DiI (magenta). Individual confocal channels, brightfield, and merged images were presented. Column Merged 1 combined the blue‐red‐green channel and column Merge 2 combined the blue‐red‐green‐magenta channel. Scale bar represents 10 μm. C57BL/6 (*n* = 8) were implanted with 1 × 10^6^ B16F10 melanoma cells on day 0, and at days 7 and 9, mice were treated intratumorally with Sol. pIpC or pIpC LNP (15 μg per injection). Tumor growth was monitored, and mice were culled at their humane endpoint (tumor diameter 15 mm). D) The mean ± SEM of tumor volume is presented in tumor growth curve. E) Statistical analysis was carried out using a Student's *T* test; mouse survival is presented in Kaplan–Meier plot, followed by Mantel–Cox test; ns, nonsignificant, *p < 0.05, ***p < 0.005.

We first validated the pIpC LNP in vitro using a THP‐1 monocytic cell line which expresses luciferase after IRF activation (THP‐1 dual). As shown in **Figure** **2**A, only the LNP formulation could induce an IRF response to pIpC. As THP‐1, in their undifferentiated state, have low expression of TLR3, this suggests that pIpC in the LNP formulation is likely reaching the cytosol and triggering RLR signaling. In keeping with this observation, we have recently demonstrated that pIpC formulation in LNP can enhance increased activation of myeloid cell subsets such as dendritic cells and macrophage.^[^ ^26^ ^]^ The uptake of the pIpC by B16F10 cells was assessed using fluorescently labeled pIpC and lipids (DiR). As shown in Figure 2B, we observed a high uptake of pIpC LNP with 70% of cells staining positive after 24 h. The pIpC signal was invariably associated with the presence of LNP, as indicated by the DiR signal. This suggests LNPs are a suitable delivery vehicle for pIpC delivery to multiple cell types. Cellular uptake of pIpC‐LNPs was also studied by confocal laser scanning microscopy (Figure 2C). B16F10 cells were incubated with DiI‐labeled and pIpC‐FL‐loaded LNP (pIpC‐FL‐LNP) for 2, 4, 8, and 24 h. The images revealed that the uptake of LNPs occurred as early as 2 h post‐treatment. Additionally, the amount of pIpC within the cytosol increased progressively from 2 to 24 h. The images suggested that LNPs are an effective system for delivering pIpC into cells, thereby enhancing its immunostimulatory potential.

To perform in vivo testing, a two‐dose intratumoral injection regime was chosen. This regime was optimized using a negative siRNA molecule (siNEG) containing LNPs. The siNEG molecule is comparable to pIpC as it is both composed of RNA and has a double‐stranded structure; however, due to its short size, it minimally engages dsRNA receptors. As shown in Figure S1, Supporting Information, IT treatment of B16F10 melanoma at days 7 and 9 with this construct has no effect on tumor growth resulting in comparable growth kinetics and survival of mice to the untreated group. When substituted for the pIpC‐LNP, tumor growth rate could be significantly reduced when compared to untreated groups or groups treated with a comparable dose of soluble pIpC (Figure 2D). Furthermore, treatment of B16F10 tumors with pIpC‐LNP resulted in survival of 25% of mice to day 50 post implantation compared to no survival for either nontreated or soluble pIpC (Figure 2E). Statistical differences however could not be obtained for survival data due to the relatively low numbers of mice surviving and group sizes. Having observed an increase in potency with LNP‐formulated pIpC, the TLR7 ligand poly uridine (pU) was likewise tested as an LNP, either alone or in combination with pIpC. Both pIpC and pU are RNA‐based molecules and thus are suitable for formulation into LNPs. As shown in Figure S2, Supporting Information, all LNP treatments resulted in reduced tumor growth; however, formulations which included pIpC were superior to pU‐LNP. When pIpC was mixed with pU (pIpC + pU) in an LNP, there was no statistical improvement compared to the pIpC‐LNP monotreatment. The potency of LNP pIpC may have been due to a number of factors including physical protection of the nucleic acid from nucleases, efficient trafficking of pIpC to the local lymph node, or more efficient delivery of pIpC to the cytosol activating RLR. While the efficacy was surprising, pIpC has been formulated with various molecules considered to be transfection reagents such as PEI and cationic liposomes; in each case, the formulation has improved the therapeutic effect in intratumoral and vaccine models.^[^ ^9^, ^27^ ^]^ The delay in tumor growth achieved by pIpC‐LNP suggests its suitability as a candidate for IT immunotherapy.

### 2.2 IT Treatment Significantly Alters T Cell Activation Markers in Tumor‐Draining Lymph Node

**Figure 3:**
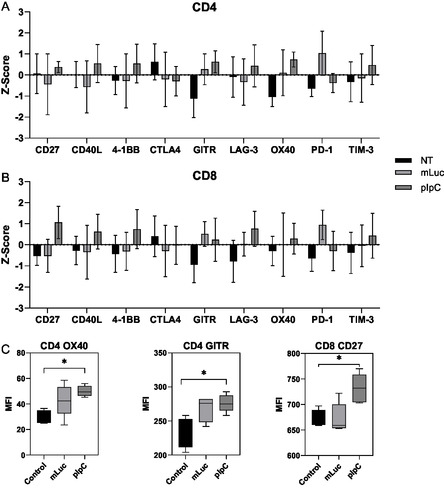
Upregulation of T cell activation markers in TDLN following IT administration of pIpC‐LNP. Mice C57BL/6 (*n *= 4) were implanted bilaterally with 1 × 10^6^ B16F10 melanoma cells on day 0, and at days 7 and 9, mice were treated intratumorally with pIpC‐LNP (pIpC) or mRNA encoding luciferase LNP (mLuc). Seventy‐two hours following the second injection, mice were culled, and TDLN were obtained. Cells were isolated from the TDLN and stained with antimouse CD3, CD4, CD8, and an antibody against an activation marker. The relative MFI of the activation marker expressed on each cell population was standardized and the *Z* score for CD4 and CD8 T cells is shown in A) and B), respectively. Based on this analysis, MFI values for selected markers are shown in C). Statistical analysis was carried out using a Student's *T* test, *p < 0.05.

Other groups have suggested that the IT administration of TLR agonists can significantly alter the expression of activation markers on T cells following treatment.^[^ ^13^, ^28^ ^]^ However, it is not known whether this is a general response to inflammation or specific to the TLR agonist utilized. Indeed, as TLR3 is unique among the TLRs, signaling solely through the TRIF intermediate, it is possible to speculate that observations made using other TLR agonists may not be comparable to pIpC. We sought to identify immune checkpoint molecules upregulated in response to pIpC LNP which may be used for synergistic targeting with nucleic acid‐based immunotherapy. To assess this, bilateral B16F10 were injected with pIpC‐LNP or an LNP containing an mRNA construct expressing luciferase (mLuc). The mLuc was used as an LNP control as this platform is routinely utilized to deliver mRNA. Three days following the second injection, cells from the tumor‐draining lymph node (TDLN) were obtained and stained with T cell phenotypic markers (CD3, CD4, CD8) plus an additional activation marker (either PD1, CTLA4, LAG3, TIM‐3, OX40, 4‐1BB, GITR, or CD27). These markers were selected based on their presence in the literature and their potential as targets for immunotherapy. They represent a mixture of both coinhibitory molecules (PD1, CTLA4, LAG3, and TIM‐3) and costimulatory members of the TNF receptor superfamily (OX40, 4‐1BB, GITR, or CD27). To assess broad changes in marker expression, rather than on specific subsets, the mean fluorescence intensity of each marker was measured for the entire CD4 T cell (CD3+, CD4+) or CD8 T cell (CD3+, CD8+) population. It should be noted that as these markers are expressed at different levels under steady states, the intensity is a relative value; as such, the data is standardized to obtain the *Z* score for each individual marker. As shown in **Figure** **3**, there were no significant changes in coinhibitory markers between groups with PD1, CTLA4, LAG3, and TIM‐3 levels remaining comparable to the control group for both CD4 and CD8 T cells. However, in the case of OX40 and GITR on CD4 T cells and CD27 on CD8 T cells, significant upregulation compared to the control was observed. These markers are plotted individually with the MFI values shown (Figure 3C). We noticed an upregulation of PD‐1 on T cells following mLuc treatment. This may be attributed to the unmodified uridine present in the mRNA backbone acting on TLR7.

Previously, it has been shown that OX40 expression is upregulated in response to CpG both in vitro and in vivo.^[^ ^13^, ^28^ ^]^ Furthermore, targeting of OX40 with an anti‐OX40 mAb results in synergistic tumor growth suppression when combined with CpG intratumorally; this synergy is also observed with TLR7/8 agonist (Resiquimod), though it was not established whether it too can upregulate OX40 expression.^[^ ^13^ ^]^ Consistent with our data on pIpC, CpG was likewise found not to alter expression of PD‐1 or CTLA4.^[^ ^13^ ^]^ Treatment of tumors with a lipophilic TLR7/8 (MED19197) agonist resulted in upregulation of not only OX40, but also costimulatory molecules GITR and 4‐1BB, and coinhibitory molecules PD1 and CTLA4 at a transcriptional level.^[^ ^29^ ^]^ When MED19197 was codelivered with anti‐OX40 mAb or GITRL fusion protein, the authors observed synergistic suppression of tumor growth.^[^ ^29^ ^]^ It may be speculated that, as OX40 and GITR are associated with regulatory T cell activity, the upregulation of these molecules may represent a general mechanism to moderate inflammation and, as such, upregulation is likely to be observed with multiple TLR agonists. We could find limited evidence of CD27 upregulation on T cells following TLR stimulation. OX40/OX40L and CD27/CD70 have been linked together in numerous studies suggesting the OX40 axis signaling is required for optimal CD4 activation, while the CD70 axis is more important in CD8 activation, though this may be an oversimplification.^[^ ^30^ ^]^

We speculate that this regulation is a bystander effect due to activation of other lymph node cell populations, or signals from the tumor rather than direct action of the pIpC on the T cells. pIpC is known to be a potent activator of dendritic cells as demonstrated in a number of models. For example, pIpC was shown to be superior to CpG or MPL (a TLR4 agonist) at inducing adaptive immune responses following a DC targeting vaccine.^[^ ^31^ ^]^ Consistent with this data, it was observed that monocyte‐derived dendritic cells matured with pIpC are superior at activating CD8 T cells compared with LPS or a cocktail of cytokines.^[^ ^32^ ^]^ In clinical trials, pIpC has been used to boost responses to autologous dendritic cell‐based cancer vaccines following infusion.^[^ ^33^ ^]^ However, TLR receptors, including TLR3, have also been identified in T cell populations at a transcriptional and protein level, regulated by T cell receptor signaling. Therefore, direct action of pIpC on T cells cannot be excluded.^[^ ^28^, ^34^ ^]^ Indeed, it has been demonstrated that the treatment of T cells with either a TLR5 or 7/8 agonists results in increased cytokine production and proliferation, an effect not seen with pIpC.^[^ ^35^ ^]^ However, direct actions of both pIpC and CpG have been shown to increase T cell survival despite not inducing proliferation.^[^ ^36^ ^]^ Combined, this data suggests that pIpC‐LNP drives the activation of T cells in the TDLN as marked by the upregulation of costimulatory molecules. In particular, CD27 and OX40 may be suitable candidates for synergistic targeting in pIpC LNP regimes.

### 2.3 Treatment of Cancer Cells with pIpC‐LNP Results in the Upregulation of PDL1 and MHC Class I

Having identified the potential T cell surface markers suitable for combined immunotherapy, direct interactions of pIpC‐LNP with B16F10 cells were next analyzed. For this, we focused on PDL1 and MHC class I, both of which have been identified as being upregulated in response to pIpC in cancer cells.^[^ ^9^, ^37^ ^]^ In addition, we have previously described upregulation of PDL1 in cancer cell lines as a response to external stressors such as cationic transfection agents complexed with pDNA, though notably not LNPs.^[^ ^38^ ^]^ If PDL1 is upregulated in response to pIpC LNP, this would represent an alternative target to those identified in the T cell screen.

**Figure 4:**
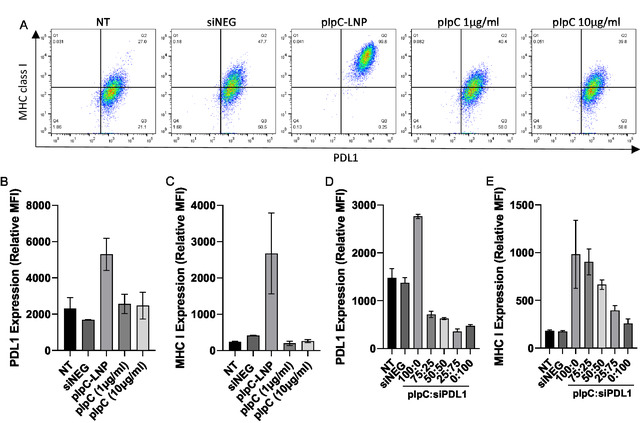
In vitro treatment of B16F10 cells with particulate pIpC results in upregulation of MHC class I and PDL1 which can be negated by inclusion of siPDL1. B16F10 melanoma cells were cultured to 70–80% confluence before being either left untreated (NT), treated with lipid nanoparticle formulated pIpC (pIpC) (1 μg mL^−1^) or soluble pIpC (at 1 μg mL^−1^ and 10 μg mL^−1^). Lipid nanoparticles containing a negative siRNA (siNEG) were used to control. Cells were harvested after 48 h of culture and stained with anti‐mouse PDL1 and MHC class I before being acquired on a FACs Celesta flow cytometer. Data analysis was performed using Flowjo software, viable cells were gated, representative flow plots are shown in A), and the relative MFI of B) PDL1 or C) MHC class I was analyzed. Alternately, cells were treated with lipid nanoparticle‐formulated pIpC (pIpC) and/or lipid nanoparticle‐formulated siPDL1 as various weight ratios. The final concentration of nucleic acid per well was fixed at 1 μg mL^−1^. After 48 h, cells were harvested, stained, and analyzed as described previously. The relative mean fluorescence intensity (MFI) of both D) PDL1 and E) MHC class I is the mean and SD of three replicates is presented.

As shown in **Figure** **4**A, untreated B16F10 expresses both PDL1, at a high level, and MHC class I, at a low level with nearly 100% and 27% of cells expressing the markers above background, respectively. Treatment with soluble pIpC at a concentration comparable to that used in the LNP (1 μg mL^−1^) or a 10‐fold excess (10 μg mL^−1^) did not increase the number of cells expressing either marker. Treatment of the cells with LNP containing siNEG did not affect the expression of either marker; however, treatment with LNP pIpC greatly increased the number of cells staining positive for both markers from 27% to 99.8% (upper right quadrant). When comparing the relative expression of each marker per cell (relative mean fluorescence intensity), pIpC LNPs approximately doubled the expression of PDL1 compared to all other treatments. Likewise, pIpC LNP substantially increased the expression of MHC I with a mean increase of 10‐fold, though it should be noted there was a high amount of variation within this dataset. Treatment of cells with pIpC‐LNP was also observed to induce apoptosis (Figure S3, Supporting Information).

From this study, we identified upregulation of PDL1 as a potential combinatory candidate. As PDL1 is associated with immune suppression, it was reasoned that the reduction of PDL1 upregulation induced by pIpC LNP may result in synergistic effects in vivo. Furthermore, upregulation of MHC class I on the cell surface may lead to increased presentation of CD8 T cell epitopes making the tumor more “immunogenic.” PDL1 upregulation following pIpC treatment has been described in a number of studies in a range of cell types including liver sinusoid endothelial cells, melanoma, glioblastoma, neuroblastoma, and breast cancer cell lines.^[^ ^37^, ^39^, ^40^, ^41^, ^42^ ^]^ Likewise, upregulation of MHC class I has also been reported including in B16 cell lines.^[^ ^9^, ^37^ ^]^ In many cases, this has been linked to the actions of type I interferons; however, we have previously reported that, at least in the case of PDL1, upregulation correlated with toxicity.^[^ ^38^ ^]^ Intriguingly, as mentioned previously, the transfection of pIpC has been shown to induce apoptosis in a number of cell lines including melanoma.^[^ ^43^, ^44^, ^45^, ^46^ ^]^ Furthermore, apoptosis may occur in the absence of type one interferon signaling.^[^ ^3^ ^]^ Whether PDL1/MHC class I upregulation is a general response to apoptosis or cell stress dependent or independent of type one interferon can only be speculated.

An LNP containing the siRNA targeting PDL1 (siPDL1) has previously been developed by our group and has been shown to be highly potent at silencing PDL1 in vitro. The siPDL1 LNP was combined with the pIpC LNP at various ratios before being added to B16F10 cells. pIpC is compatible with siRNA and has previously been formulated with siSTAT3 and siPDL1 in vaccination models.^[^ ^47^, ^48^ ^]^ As shown in Figure 4B, PDL1 upregulation following treatment with LNPs encapsulating pIpC, presenting the 100% plpC dose, was consistently observed with increasing expression from an average of 1479 to 2760 relative MFI. Although it should be noted that the data presented in Figure 4B–E are independent of each other, and the difference in MFIs reflects experimental variation, as such the panels are not directly comparable. Replacing 25% of the pIpC LNPs with LNPs containing siPDL1 (ratio 75:25), as separate formulations, reduced PDL1 levels to below basal levels of expression (MFI: 721). Increasing the quantities of PDL1 LNPs showed little additional benefit, with 100% PDL1 LNPs lowering the MFI to 470. Upregulation of MHC class I was observed in all cells receiving LNP pIpC; however, starting from 75:25 (pIpC:siPDL1), a dose‐dependent decrease in upregulation was observed. To maximize MHC class I expression and minimize PDL1 expression, a ratio of 75:25 pIpC:siPDL1 was selected as a combinatory candidate.

### 2.4 Evaluation of Potential T Cell Targets for Synergistic Combination with pIpC LNP

**Figure 5:**
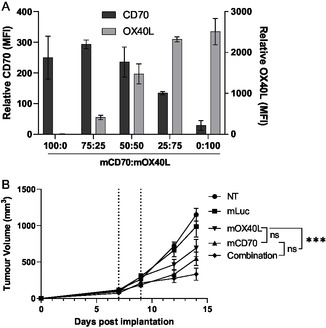
B16F10 cells can be cotransfected with multiple mRNA constructs and the combination of mRNAs results in suppression of tumor growth. A) B16F10 melanoma cells were transfected with lipid nanoparticles containing mRNAs expressing either OX40L and/or CD70 at various weight ratios as described. The final concentration of mRNA was set to 1 μg mL^−1^. Cells were harvested after 48 h transfection and costained with antimouse CD70 and antimouse OX40L, before being acquired on a FACs Celesta flow cytometer. Cells were also stained with corresponding isotype control antibodies. Data was analyzed using FlowJo software by first gating on the viable cells, then measuring the Mean fluorescence intensity (MFI). Data presented represents the average isotype‐subtracted MFI of three replicates with SD. B) B16F10 cells were implanted into C57BL/6 (*n* > 7) on day 0; at days 7 and 9 postimplantation, tumors were injected intratumorally with LNPs containing mRNA encoding Luciferase (mLuc), OX40L, CD70, or a combination of OX40L and CD70 (Combination). In each case, a total of 7.5 μg mRNA per dose was used, and an additional group of mice was left untreated (NT). Tumors were measured at intervals of 2–3 days. The mean ± SEM tumor volume is plotted in each case. Statistical analysis was carried out with a two‐way ANOVA; ns, nonsignificant, ***p < 0.005.

As outlined previously, we identified a number of tumor necrosis superfamily receptor (TNFSF) molecules are upregulated in the TDLN following treatment with pIpC. To complement this upregulation, LNPs containing mRNA encoding their cognate ligand were prepared. Two candidates were selected OX40L and CD70; the mRNAs encoding these molecules were correspondingly named mOX40L and mCD70. This selection was not only based on our preliminary data, but also our own and others’ description of mOX40L as a candidate for intratumoral therapy and its reported synergy with TLR ligands. CD70 was selected as its receptor, CD27, was the only molecule found to be upregulated on CD8 T cells (Figure 3), though it is less prominent in the literature. The mRNAs were synthesized, formulated into LNPs as previously described, and used to transfect B16F10 cells in vitro. As shown in **Figure** **5**A, low to no CD70 or OX40L expression was detected on B16F10 cells as indicated by the minimal signal in conditions which did not receive the corresponding mRNA (i.e., no detectable signal from OX40L in the 100% mCD70 group). When LNPs were applied, we could achieve cotransfection with both CD70 and OX40L with no detectable interference. Expression levels correlated with the quantity of mRNA LNPs added with CD70 expression at an average MFI of 200–300 when LNPs were used at 100–50% of total LNPs, dropping to an MFI of 120 at 25%. OX40L followed a similar trend, with expression levels between 2000 and 3000 at 75–100%, dropping to MFIs of 1474 and 410 for 50:50 and 75:25, respectively. The MFI values obtained for OX40L expression were considerably higher than those obtained for CD70; these represent relative values only. Direct comparison between OX40L and CD70 cannot be performed as these molecules were stained with different fluorophore‐conjugated mAbs and distinctions may represent the quantum yield of the fluorophore. For future combinational studies, a 50:50 OX40L:CD70 LNP ratio was selected. We have previously demonstrated that mRNA formulated to this specification is expressed when delivered intratumorally using mLuc; however, we have also shown that direct assessment of immune checkpoint expression/knockdown is hampered by the rapid elimination of modified cells.^[^ ^21^, ^49^ ^]^

To test for any in vivo synergistic effects between mCD70 and mOX40L, B16F10 bearing mice were injected IT twice in a regime comparable to the one utilized in Figure 2. A total dose of 7.5 μg mRNA was used in all cases to allow for slight differences to be observed; furthermore, as immunogenic, unmodified mRNA was used, we wanted the effects to be specific to the transgene. As shown in Figure 5B, tumor growth was consistent between experiments with untreated tumors reaching 1100 mm^3^ at day 14 postimplantation. Treatment with the negative control mRNA (mLuc) did not statistically affect tumor growth. In all groups receiving mRNAs encoding costimulatory molecules, there was significant inhibition of tumor growth compared to untreated and mLuc‐treated. mCD70‐mOX40L LNPs resulted in the highest reduction in tumor volume and as such were selected to progress in combination with pIpC LNP.

From the above data, we identified two approaches to potentially improve the therapeutic effect of pIpC LNP using codelivery of nucleic acid. In the first approach, pIpC LNP will be delivered alongside an mRNA LNP (expressing mCD70 and mOX40L); it was hypothesized that the expression of these molecules will aid in the potentiation of inflammation induced by pIpC by promoting a T cell response. In the second approach, pIpC LNP will be delivered with a siPDL1 LNP to reduce the immunosuppressive nature of the tumor microenvironment.

### 2.5 Combinatory Therapy with LNPs Containing mRNA Does Not Improve the Therapeutic Effect of pIpC LNP

In the preliminary studies, we identified that CD27 and OX40 are upregulated following LNP pIpC treatment; furthermore, their ligands, CD70 and OX40L respectively, can be coexpressed resulting in vitro and exert a therapeutic effect in vivo. The use of mRNA to deliver immunostimulatory agents, rather than mAbs raised against the receptor, is becoming increasingly popular in the literature. Particularly, the delivery of mOX40L has been shown to be efficacious in numerous preclinical studies, while the delivery of mRNA encoding CD70 has been less effective.^[^ ^18^, ^19^ ^]^ Nevertheless, both were selected as an approach to target CD4 and CD8 T cells, respectively. The mRNA LNPs were next tested in an in vivo B16F10 model including pIpC LNPs in the dosage regimen. In keeping with the previous work, a two‐dose regime was selected using 15 μg of nucleic acid to match the initial pIpC study (Figure 2). As we used native non‐nucleoside modified mRNA, by matching mRNA and pIpC doses, the intrinsic immunogenicity of the mRNA relative to the pIpC will be accounted for, increasing the validity of the conclusion. However, in contrast to previous studies, a heterologous dosing regime was utilized, with the pIpC LNPs either being administered 2 days preceding or following the LNP containing mCD70 and mOX40L (mRNA), resulting in 4 treatment groups: pIpC‐pIpC, pIpC‐mRNA, mRNA‐pIpC, mRNA‐mRNA. This regime was selected as the costimulatory molecule upregulation was identified 2 days post‐treatment; it was reasoned that a time interval between doses may be required for the T cells to respond to the LNP pIpC. We found the presence of pIpC did not significantly affect mRNA expression in vitro or in vivo (Figure S4, Supporting Information). In the case of in vivo expression, modified mRNA was utilized to eliminate any type one interferon responses elicited by the mRNA itself.

**Figure 6:**
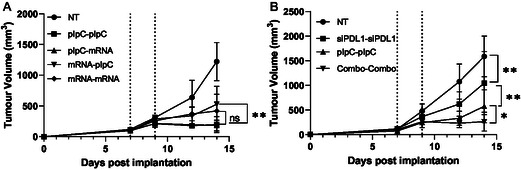
Targeting positive immune checkpoints does not significantly improve on a pIpC regime; however, targeting negative checkpoints has a minor benefit. C57BL/6 mice (*n* = 8) were implanted with B16F10 melanoma cells. Tumors were allowed to form and, at days 7 and 9 postimplantation, were treated intratumorally with LNP. In the case of A), mice received either a homologous pIpC or mRNA LNP (mCD70/OX40L 50:50) at days 7 and 9 (pIpC‐pIpC and mRNA‐mRNA, respectively) or a heterologous regime comprising either pIpC or mRNA LNP at days 7 and 9 (pIpC‐mRNA and mRNA‐pIpC). Alternately, in the case of B), two homologous doses of LNPs incorporating either siPDL1, pIpC, or a mixture of the two at pIpC:siPDL1, 75:25 ratio, Combo, were administered. A total of 15 μg mRNA per dose was used. In each study, a group of mice was untreated (NT). Tumors were measured at intervals of 2–3 days. The mean ± SD tumor volume is plotted in each case. Statistical analysis was carried out using a Student's *T* test (*p < 0.05) or two‐way ANOVA (*p < 0.01); ns, nonsignificant.

As shown in **Figure** **6**A, all treatments resulted in significant growth reduction compared to the nontreated control. Consistent with our previous data, groups receiving pIpC LNP firstly pIpC‐pIpC and pIpC‐mRNA had the lowest end tumor volume (mean volume 182 mm^3^) with no difference detected between pIpC‐pIpC and pIpC‐mRNA groups. This volume was statistically different from the mRNA‐pIpC (527 mm^3^) but not the mRNA‐mRNA (418 mm^3^) group. No significant difference between mRNA‐pIpC and mRNA‐mRNA was observed. This highlights the high potency of pIpC when delivered as an LNP early in a therapeutic regime suggesting its suitability as a standalone candidate. Additionally, results suggest the need for a repeated mRNA dosing regimen for therapeutic efficacy improvement compared to pIpC LNP.

### 2.6 Combinatory Therapy with LNPs Containing siRNA Results in Modest Improvements to the Therapeutic Effect of pIpC LNP

Combinatory LNP formulations designed to target the tumor cells and immunosuppressive microenvironments were next tested. As PDL1 was identified as being upregulated following treatment of cancer cells with pIpC LNP, siPDL1 LNPs were coadministered simultaneously as separate LNPs. The four treatment groups are untreated, siPDL1‐siPDL1, pIpC‐pIpC, or siPDL1/pIpC‐siPDL1/pIpC (combination group) at a total NA dose of 15 μg repeated twice at 2 day intervals. In the combination group, siPDL1 LNPs were coadministered at the same time as plpC‐LNPs since unlike mRNA, type one interferon is not known to affect the action of siRNA. pIpC:siPDL1 dose ratio was 75:25 mimicking in vitro studies.

As shown in Figure 6B, the greatest effect was observed with LNPs incorporating pIpC and siPDL1 (combo) (276 mm^3^), followed by pIpC‐pIpC (576 mm^3^), then siPDL1‐siPDL1 (1047 mm^3^) group compared to 1588 mm^2^ volume in the control group. PDL1 blockade has been previously used alongside pIpC and has shown synergistic tumor growth suppression.^[^ ^8^, ^50^ ^]^ The differences between our study and those of others are the use of mRNA/siRNA rather than mAbs.

### 2.7 Characterization of Tumor and TDLN Cell Populations Following Treatment with LNP pIpC

**Figure 7:**
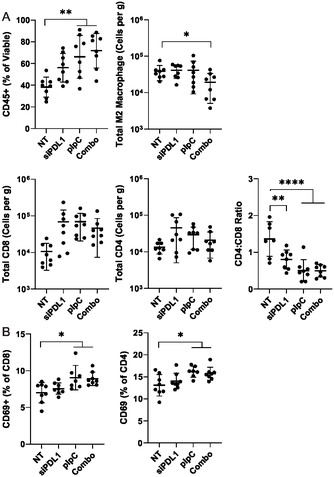
Delivery of pIpC and siPDL1 significantly alters tumor and TDLN leukocyte populations. Following treatment of tumors with LNPs containing either siPDL1, pIpC, or a mixture of the aforementioned (Combo) (15 μg nucleic acid per dose) on days 7 and 9 post‐tumor implantation, mice were culled (day 14 post implantation). Cells were isolated from tumor and TDLN via physical dissociation and stained with relevant antimouse fluorophore‐conjugated monoclonal antibodies. A) The following cell populations in the tumor were analyzed: total leukocytes (CD45+), M2 macrophage (CD45+, CD11b+, F480+, CD206+), CD8 T cells (CD45+, CD3+, CD8+), CD4 T cells (CD45+, CD3+, CD4+). B) To analyze T cell activation in the TDLN cells were stained with the aforementioned T cell markers and activation marker (CD69+). Cells were acquired on a FACs Celesta flow cytometer, and flow cytometric analysis was performed using Flowjo software. In each case, mean ± SD is plotted. Statistical analysis was performed using Student's *T* test or one‐way ANOVA *p < 0.05, **p < 0.01, ****p < 0.0001, ns nonsignificant.

Having observed highly potent tumor growth suppression with pIpC LNP, which can be improved with the inclusion of siPDL1, the effect of these formulations on tumor and TDLN cell populations was characterized. It was observed in **Figure** **7** that in groups receiving pIpC (pIpC‐pIpC or pIpC/siPDL1‐pIpC/siPDL1), leukocytes constitute a mean of 60–70% of cells compared to the nontreated control of which only 40% of cells were leukocytes. Furthermore, there was a significant reduction in the pro‐tumorigenic M2 macrophage population in combo groups and not any of the other groups, which remains unexplained. This study focused on M2 macrophages as these are typically present in the later stages of tumor progression and a reduction in their incidence may be indicative of better tumor control.^[^ ^51^ ^]^

In terms of T cell populations, there was a trend toward increasing numbers of T cells in groups receiving LNP formulations, though this did not achieve statistical significance. To determine whether there was a bias in T cell phenotype, the ratio of CD4:CD8 was plotted, with higher values indicating a CD4 skewing. As shown in Figure 7A, all LNP groups had significant skewing toward a CD8 T cell response with the mean number of CD4 T cells per CD8 T cells being less than one, compared with the nontreated group which has an average of 1.3 CD4 to CD8 ratio. This effect was most pronounced in groups receiving pIpC or combo LNP, with a combined mean of the two groups averaging two CD8 T cells per CD4 T cell. There were no significant differences between the two groups receiving pIpC LNPs. When the TDLN was analyzed, it was observed that in groups receiving pIpC or combo LNPs, there was a slight but significant increase in the number of cells expressing the early activation marker CD69 in both CD4 and CD8 subsets compared to nontreated control, suggesting ongoing activation. Future work will focus on identifying the definitive mechanism of pIpC‐LNP, including the key cellular players involved in tumor growth suppression.

## 3 Conclusions

**Scheme 2:**
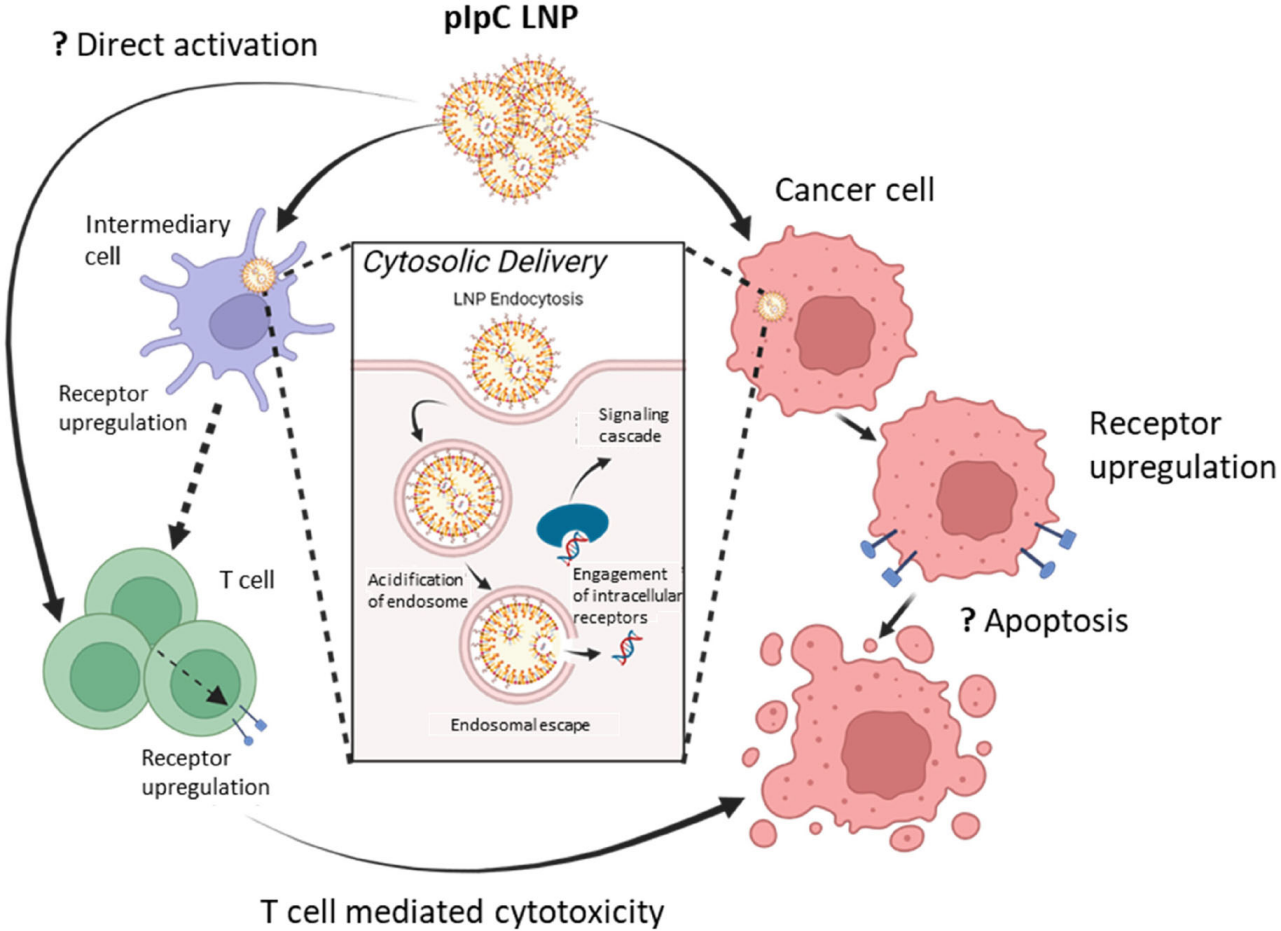
Proposed mechanism for pIpC‐LNP. Following intratumoral injection, pIpC‐LNP may be endocytosed by both cancer cells and leukocytes such as antigen‐presenting cells. The LNP system facilitates endosomal escape of pIpC to the cytosol. Once in the cytosol, pIpC can engage with specific receptors leading to a signaling cascade. In cancer cells, this results in upregulation of PDL1 and MHC class I and potentially induces apoptosis. pIpC signaling also leads to activation of T cells in the TDLN either directly or indirectly through an intermediary cell. T cell activation is marked by an upregulation of various receptors including GITR, CD27, and OX40. It is proposed that T cell activation can then induce T cell‐mediated cytotoxicity of cancer cells. T cell expressed receptors and tumor expressed ligands represent potential targets for combinatory nucleic acid immunotherapy; this study supports the cotargeting of PDL1.

We have developed a pIpC formulation based on the clinically acceptable ionizable LNP platform. We have demonstrated that, when administered intratumorally, this formulation potently suppresses tumor growth and activates T cells as proposed in **Scheme** **2**. Furthermore, we have demonstrated that the platform is compatible with codelivery of mRNA and siRNA. In this study, combining siPDL1 with pIpC resulted in improved therapeutic efficacy compared to pIpC monotherapy. Combined, these data support the development of pIpC‐LNP for clinical application. Future work may consist of the optimization of the LNP pIpC regime with an anti‐PDL1, OX40, GITR, and CD27 mAb cocktail.

## 4 Experimental Section

4.1

4.1.1

##### Materials

Molecular biology kits and reagents, XbaI, HiScribe T7 ARCA mRNA Kit (with tailing)TM, Monarch RNA clean up kitTM, pseudo‐UTP, 5‐mCTP, and Monarch PCR&DNA clean up kitTM were purchased from New England Biolabs. Quant‐itTM Ribogreen was purchased from Thermofisher. Amicon Ultra 30KDa MWCO filter units were obtained from Merck Millipore. siRNA duplexes were purchased from Eurogentec (siPDL1 sequence GAGGUAAUCUGGACAAACA; siNEG sequence UGCGCUACGAUCGACGAUG). Plasmids containing the murine OX40L ORF (TNFSF4 ORF) and murine CD70 (TNFSF7 ORF) were obtained from Sinobiological. Luciferase‐pcDNA3 plasmid was a gift from William Kaelin (Addgene plasmid # 18 964). Polyinosinic:polycytidylic acid (pIpC) was purchased from Bio‐techne; polyuridylic acid (pU) and fluorescein‐labeled pIpC (pIpC‐FL) were purchased from Invivogen. 1,1′‐Dioctadecyl‐3,3,3′,3′‐tetramethylindocarbocyanine perchlorate (DiI; M16349) and Hoechst 33342 (M84002) were purchased from Meryer. LysoTracker Red (C1046) was purchased from Beyotime. 300 mesh carbon‐coated copper grids (BZ11023a) were purchased from Zhongjingkeyi Technology. Phosphotungstic acid (PTA) negative staining solution (2%; PH1308) was purchased from Phygene. Lipids utilized included 1,2‐dioleoyl‐sn‐glycero‐3‐phosphoethanolamine (DOPE) (Lipoid), cholesterol (Sigma–Aldrich), Dlin‐MC3‐DMA (Bioybt), and N‐palmitoyl‐sphingosine‐1‐succinyl [methoxy (polyethylene glycol) 2000] (C16 PEG 2000 Ceramide) (Avanati). Anti‐mouse fluorophore conjugated antibodies (CD8‐PE (53‐6.7), CD4‐FITC/PE (GK1.5), PDL1‐PE (10F.9G2), OX40L‐APC/PE (RM134L), CD70‐PE/APC (FR70), CD69‐APC/APC‐Cy7 (H1.2F3), CD3‐APC/PerCP (17A2), CD45‐PerCP (30‐F11), MHC class I‐APC (AF6‐88.5), F480‐FITC, (QA17A29), CD11b‐PE (M1/70), CD206‐APC (C068C2), PD1‐APC (29F.1A12), CTLA4‐APC (UC10‐4B9), LAG3‐APC (C9B7W), OX40‐APC (OX‐86), 4‐1BB‐APC (17B5), GITR‐APC (DTA‐1), CD27‐APC (LG.3A10)), Zombie AquaTM fixable viability dye, TruStain FcXTM PLUS, Precision count beadsTM, and Annexin V kit were purchased from BioLegend. Anti‐mouse TIM‐3‐APC (RMT3‐23) was purchased from eBioscience. All antibodies were used at a 1:200 dilution. For multicolor flow cytometry, UltraComp ebeadsTM and ARC Amine Reactive beads (both Thermofisher) were used to perform compensation. Tissue culture reagents: fetal calf serum (FCS), trypsin EDTA, GlutaMAXTM, RPMI 1640 media, phosphate buffer saline (PBS), and penicillin‐streptomycin were from Gibco, Thermo Fischer Scientific. Quanti‐Luc, Zeocin, and Blasticidin were purchased from Invivogen. Unless stated otherwise, all chemical reagents were purchased from Sigma–Aldrich.

##### Formulation and Characterization of Nucleic Acid Lipid Nanoparticles

Nucleic acid was incorporated into LNPs using a ethanol dilution method;^[^ ^52^ ^]^ in brief, an acidified ethanolic solution of lipids (Dlin‐MC3‐DMA, cholesterol, DOPE and C16 ceramide PEG 2000 at a molar ratio of 35:46.5:16:2.5 respectively) was gradually added to a solution of nucleic acid in 20 mM citrate buffer pH4 at intervals of 10% v/v of nucleic acid solution, before being vigorously vortexed for 30 s and incubated for a further 30 s. The mass ratio of nucleic acid to ionizable lipid (Dlin‐MC3‐DMA) was maintained at 1:10. Following the final addition of lipid solution, LNPs were incubated for 1 h at 37 °C before excess ethanol was evaporated under a dry stream of nitrogen. For use in vivo, LNPs were further concentrated using an Amicon Ultra 30KDa MWCO filter unit by centrifugation (12 000 rpm for 30 min at 4 °C, Eppendorf) and buffer exchanged to 20 mM HEPES buffered saline pH 7.2. Physical parameters were measured using dynamic light scattering. LNP suspension was diluted in 0.1X PBS before being added to a folded capillary zeta cell and run on a Zetasizer Nano (Malvern Instruments). All measurements represent an average of 20 runs; each run was completed in triplicate at 25 °C. Encapsulation efficiency of nucleic acid was determined by Ribogreen assay as described elsewhere.^[^ ^53^ ^]^ LNPs were named according to their nucleic acid content.

##### Transmission Electron Microscopy with Negative Staining

Copper grids were glow discharged for 60 s using PELCO easiGlow Glow Discharge Cleaning System. 2.5 μL of pIpC‐LNP solution was added onto the glow‐discharged side for 120 s and carefully blotted with a filter paper. 2% PTA solution was then added onto the grid for 20 s and carefully blotted with a filter paper. The grids were imaged by Talos L12C TEM (ThermoFisher) with 120 kV at cryogenic temperature. Images were with Ceta CMOS camera with Velox Software.

##### mRNA Synthesis

Commercial plasmids encoding OX40L, CD70, or luciferase were linearized using XbaI restriction enzyme according to protocol and purified using a DNA extraction kit. Non‐nucleotide modified mRNA was synthesized via in vitro transcription with a HiScribe T7 ARCA mRNA synthesis kit with tailing according to the manufacturer's protocol and purified using an RNA purification kit. Synthesized mRNA was quantified by UV absorbance using a Nano Drop one (Thermofisher). For mLuc in vivo study, pseudo‐UTP and 5‐mCTP were added to the in vitro transcription reaction.

##### Cell Culture

B16F10 were grown in a humidified 37 °C, 5% CO_2_ incubator and maintained in DMEM medium supplemented with 1% v/v Glutamax, 50 U/mL penicillin, 10 μg mL^−1^ streptomycin, and 10% FCS. Cells were passaged at 90% confluence by first removing spent media, then washing cell sheet with PBS. Cells were disassociated by incubation with 0.05% trypsin EDTA at 37 °C followed by neutralization with culture media; cells were typically split at a 1:5‐1:10 ratio 2‐3 times per week. THP‐1 dual cells were purchased from InvivoGen and cultured in RPMI 1640 medium supplemented with 10% heat‐inactivated FBS and selective antibiotics Zeocin (100 μg mL^−1^) and Blasticidin (10 μg mL^−1^).

##### THP‐1 Dual IRF Activation Assay

THP‐1 Dual cells were seeded at 100 000 cells/well in 96‐well plates and treated with for 24 h. Cell supernatant (20 μL) was transferred to an opaque 96‐well plate and luciferase in the supernatant was measured using a QUANTI‐Luc reagent (50 μL) reconstituted to the manufacturer's specification. Luminescence was measured immediately using an EnVision plate reader (PerkinElmer).

##### In Vitro Transfection/Stimulation

The day prior to transfection/stimulation, B16F10 cells were plated in 12‐well plates at a density of 100 K cells per well in 1 mL of tissue culture media to obtain 90% confluence. The following day, LNP or soluble pIpC was diluted in tissue culture media before being added directly to the corresponding wells to obtain the desired final concentration. Plates were incubated for 48 h under cell culture conditions. Cells were harvested from plates by first removing media, washing with PBS, then incubating with 0.05% trypsin EDTA. Harvested cells were washed with PBS before being stained with relevant antimouse fluorophore conjugated monoclonal antibody. In brief, cells were suspended in 100 μL of PBS before 50 μL of prediluted monoclonal antibody solution was added. Cells were incubated for 30 min at 4 °C before excess, unbound antibody was washed off by the addition of 200 μL PBS and centrifugation (1750 rpm, 4 °C 5 min, Eppendorf). Washing was repeated three times. Stained cells were acquired on a FACs Celesta flow cytometer (BD Biosciences). Annexin V staining was carried out in accordance with the manufacturer's datasheet; cells were suspended in 1X annexin V binding buffer before being incubated with FITC‐conjugated annexin for 30 min at 4 °C; cells were then washed as described above; 7AAD was added and incubated for 15 min. Cells were then directly acquired on a FACs Celesta flow cytometer (BD Biosciences). Data analysis was performed using FlowJo software (Treestar). Data is presented either as mean fluorescence intensity (MFI) of the marker of interest or the percentage of cells staining positive above background. To measure luciferase expression following transfection with mLuc, cells were lysed by repeated freeze thawing at −80 °C. Cell lysate was then clarified by centrifugation at 14 000 rpm for 5 min; supernatant was taken and mixed with luciferin substrate. Luminescence was measured on a BMG LABTECH, FLUOstar Omega plate reader. The protein in the cell extract was measured using a BCA protein assay kit according to the manufacturer's data sheet. Data was normalized by dividing luciferase signal by protein quantity.

##### Confocal Microscopy

B16F10 cells were seeded at a density of 10 000 cells/well in a 96‐well plate and allowed to adhere overnight. Cells were then treated with 0.1 μL of DiI‐labeled LNPs (0.5 mol% of lipid) encapsulating 0.4 μg pIpC‐FL (4 μg mL^−1^) for 2, 4, 8, and 24 h. After treatment, cells were stained with LysoTracker Red (100 nM) for 60 min and Hoechst 33342 for 5 min before imaging. Images were acquired by Zeiss LSM900 confocal laser scanning microscope with a plan apochromat 40×/1.4 Oil DIC objective and analyzed by ZEISS Zen software.

##### Animals

Animal experiments were conducted with project (PPL PP8950634) and personal licenses granted by the UK Home Office and in accordance with the UKCCCR Guidelines (1998). Female C57BL/6 mice of 4–6 weeks in age were obtained from Charles River.

##### In Vivo Tumor Growth and Immunogenicity Studies

C57BL/6 mice were implanted with 1 × 10^6^ B16F10 cells suspended in 100 μL PBS subcutaneously into the right flank. At days 7 and 9 postimplantation, tumors were injected with either LNP formulation or soluble adjuvant at 7.5‐15 μg per dose in 50 μL PBS. Tumors were measured with a digital calliper every 2–3 days. For growth curve analysis, all mice were culled at day 14 postimplantation. At the termination of the study, mice were sacrificed by cervical dislocation and tissues extracted for further analysis. In survival studies, mice were continually monitored until they had reached their predetermined humane endpoint: tumor size exceeding 14 mm in diameter, greater than 15% weight loss, or visual signs of distress, at which point they were sacrificed by cervical dislocation.

##### Assessment of Immunological Response Following Therapy

Postmortem murine tumor and TDLN (inguinal) were physically macerated with the flat end of a syringe plunger into a 12‐well tissue culture plate, washed with PBS, and passed through a 45 μm cell strainer to obtain a single‐cell suspension. Cells were centrifuged and washed with PBS twice before being resuspended in PBS. Cells were first incubated with Zombie Aqua viability dye before phenotypic surface markers were stained with antimouse fluorophore‐conjugated monoclonal antibodies; cells were then washed three times and fixed with 4% paraformaldehyde. TruStain FcX PLUS was added to each stain to block antibody interaction with Fc receptor. Prior to acquisition, precision count beads were added to the sample to enable cell counting. Events were acquired on FACs Celesta flow cytometer (BD Biosciences) or a FACs Calibur (BD Biosciences). Data analysis was performed using the FlowJo software package. Markers utilized in each study are outlined in the corresponding figure legend (CD8 T cells, CD3+, CD8+; CD4 T cells, CD3+, CD4+; activated T cells as previously described plus CD69+; M2 macrophage, CD45+, CD11b+, F480+, CD206+), cells were first gated based on forward/side scatter profile followed by singlet identification (forward scatter high versus area), and viable cells were identified based on Zombie Aqua staining. In each case, data is presented as either the total number of cells per TDLN/gram of tumor or the percentage of the parent population.

In the case of TDLN phenotyping studies, T cells (CD3+, CD4/CD8+) were stained as described above; however, an additional antibody specific for the marker of interest was added (including PD1, CTLA4, LAG3, TIM‐3, OX40, 4‐1BB, GITR, or CD27). T cells were gated and the MFI of the marker of interest was assessed. For illustrative purposes, data were standardized to obtain a Z score.

##### Statistical Analysis

Statistical analysis was carried out using the Graphpad Prism 9 software package. Unless stated otherwise, data are presented as mean ± SD (standard deviation) of three replicates representative of two to three experimental repeats. Tumor growth is expressed as mean ± SEM (standard error of the mean) of *n* => 5 animals per group. To compare two parameters, a Student's *t*‐test was utilized; for greater than two parameters, an ANOVA followed by relevant post‐test was performed. The statistical test utilized and statistically significant differences, denoted by an asterisk, are noted in the figure legend.

## Supporting Information

Supporting Information is available from the Wiley Online Library or from the author.

## Conflict of Interest

Amer F. Saleh is currently an employee of AstraZeneca and may or may not own stock options in the company.

## Supporting information

Supplementary Material

## Data Availability

The data that support the findings of this study are available from the corresponding author upon reasonable request.

## References

[1] Y. S. Cheng , F. Xu , Cancer Biol. Ther. 2010, 10, 1219. 20930504 10.4161/cbt.10.12.13450

[2] M. Matsumoto , T. Seya , Adv. Drug Deli. Rev. 2008, 60, 805. 10.1016/j.addr.2007.11.005 18262679

[3] R. Besch , H. Poeck , T. Hohenauer , D. Senft , G. Hacker , C. Berking , V. Hornung , S. Endres , T. Ruzicka , S. Rothenfusser , G. Hartmann , J. Clin. Invest. 2009, 119, 2399. 19620789 10.1172/JCI37155 PMC2719920

[4] T. Nakano , E. T. Yamamura , H. Fujita , T. Sone , K. Asano , Biosci. Biotechnol. Biochem. 2018, 82, 1889. 30079840 10.1080/09168451.2018.1501264

[5] N. Hilf , S. Kuttruff‐Coqui , K. Frenzel , V. Bukur , S. Stevanovic , C. Gouttefangeas , M. Platten , G. Tabatabai , V. Dutoit , S. H. van der Burg , P. T. Straten , F. Martínez-Ricarte , B. Ponsati , H. Okada , U. Lassen , A. Admon , C. H. Ottensmeier , A. Ulges , S. Kreiter , A. von Deimling , M. Skardelly , D. Migliorini , J. R. Kroep , M. Idorn , J. Rodon , J. Piró , H. S. Poulsen , B. Shraibman , K. McCann , R. Mendrzyk , et al., Nature 2019, 565, 240. 30568303 10.1038/s41586-018-0810-y

[6] L. Hammerich , T. U. Marron , R. Upadhyay , J. Svensson‐Arvelund , M. Dhainaut , S. Hussein , Y. Zhan , D. Ostrowski , M. Yellin , H. Marsh , A. M. Salazar , A. H. Rahman , B. D. Brown , M. Merad , J. D. Brody , Nat. Med. 2019, 25, 814. 30962585 10.1038/s41591-019-0410-x

[7] C. Y. Wu , H. Y. Yang , A. Monie , B. Ma , H. H. Tsai , T. C. Wu , C. F. Hung , Cancer Immunol., Immunother.: CII 2011, 60, 1085. 21526359 10.1007/s00262-011-1013-7 PMC4631404

[8] I. Marquez‐Rodas , F. Longo , M. E. Rodriguez‐Ruiz , A. Calles , S. Ponce , M. Jove , B. Rubio‐Viqueira , J. L. Perez‐Gracia , A. Gomez‐Rueda , S. Lopez‐Tarruella , M. Ponz‐Sarvise , R. Alvarez , A. Soria‐Rivas , E. de Miguel , R. Ramos‐Medina , E. Castanon , P. Gajate , C. Sempere‐Ortega , E. Jimenez‐Aguilar , M. A. Aznar , A. Calvo , P. P. Lopez‐Casas , S. Martin‐Algarra , M. Martin , D. Tersago , M. Quintero , I. Melero , Sci. Transl. Med. 2020, 12, 565, 10.1126/scitranslmed.abb0391. 33055241

[9] M. A. Aznar , L. Planelles , M. Perez‐Olivares , C. Molina , S. Garasa , I. Etxeberria , G. Perez , I. Rodriguez , E. Bolanos , P. Lopez‐Casas , M. E. Rodriguez‐Ruiz , J. L. Perez‐Gracia , I. Marquez‐Rodas , A. Teijeira , M. Quintero , I. Melero , J. Immunother. Cancer 2019, 7, 116. 31046839 10.1186/s40425-019-0568-2 PMC6498680

[10] M. Alvarez , C. Molina , C. E. De Andrea , M. Fernandez‐Sendin , M. Villalba , J. Gonzalez‐Gomariz , M. C. Ochoa , A. Teijeira , J. Glez‐Vaz , F. Aranda , M. F. Sanmamed , M. E. Rodriguez‐Ruiz , X. Fan , W. H. Shen , P. Berraondo , M. Quintero , I. Melero , J. Immunother. Cancer 2021, 9, e002953, 10.1136/jitc-2021-002953. 34824158 PMC8627419

[11] A. Ribas , T. Medina , S. Kummar , A. Amin , A. Kalbasi , J. J. Drabick , M. Barve , G. A. Daniels , D. J. Wong , E. V. Schmidt , A. F. Candia , R. L. Coffman , A. C. F. Leung , R. S. Janssen , Cancer Disc. 2018, 8, 1250. 10.1158/2159-8290.CD-18-0280 PMC6719557 30154193

[12] Z. Zhou , L. Lin , Y. An , M. Zhan , Y. Chen , M. Cai , X. Zhu , L. Lu , K. Zhu , J. Hepatocell. Carcinoma. 2021, 8, 529. 34136421 10.2147/JHC.S301375 PMC8197594

[13] I. Sagiv‐Barfi , D. K. Czerwinski , S. Levy , I. S. Alam , A. T. Mayer , S. S. Gambhir , R. Levy , Sci. Transl. Med. 2018, 10, 426, 10.1126/scitranslmed.aan4488. PMC5997264 29386357

[14] M. Schlich , R. Palomba , G. Costabile , S. Mizrahy , M. Pannuzzo , D. Peer , P. Decuzzi , Bioeng. Transl. Med. 2021, 6, e10213. 33786376 10.1002/btm2.10213 PMC7995196

[15] X. Han , H. Zhang , K. Butowska , K. L. Swingle , M. G. Alameh , D. Weissman , M. J. Mitchell , Nat. Commun. 2021, 12, 7233. 34903741 10.1038/s41467-021-27493-0 PMC8668901

[16] L. J. Kubiatowicz , A. Mohapatra , N. Krishnan , R. H. Fang , L. Zhang , Explor. Beijing 2022, 2, 20210217. 10.1002/EXP.20210217 PMC9539018 36249890

[17] S. Shirai , M. Shibuya , A. Kawai , S. Tamiya , L. Munakata , D. Omata , R. Suzuki , T. Aoshi , Y. Yoshioka , Front. Immunol. 2019, 10, 3018. 31998305 10.3389/fimmu.2019.03018 PMC6962196

[18] O. A. W. Haabeth , T. R. Blake , C. J. McKinlay , A. A. Tveita , A. Sallets , R. M. Waymouth , P. A. Wender , R. Levy , Cancer Res. 2019, 79, 1624. 30692215 10.1158/0008-5472.CAN-18-2867 PMC6445668

[19] S. L. Hewitt , A. Bai , D. Bailey , K. Ichikawa , J. Zielinski , R. Karp , A. Apte , K. Arnold , S. J. Zacharek , M. S. Iliou , K. Bhatt , M. Garnaas , F. Musenge , A. Davis , N. Khatwani , S. V. Su , G. MacLean , S. J. Farlow , K. Burke , J. P. Frederick , Sci. Transl. Med. 2019, 11, 477, 10.1126/scitranslmed.aat9143. 30700577

[20] C. Hotz , T. R. Wagenaar , F. Gieseke , D. S. Bangari , M. Callahan , H. Cao , J. Diekmann , M. Diken , C. Grunwitz , A. Hebert , K. Hsu , M. Bernardo , K. Kariko , S. Kreiter , A. N. Kuhn , M. Levit , N. Malkova , S. Masciari , J. Pollard , H. Qu , S. Ryan , A. Selmi , J. Schlereth , K. Singh , F. Sun , B. Tillmann , T. Tolstykh , W. Weber , L. Wicke , S. Witzel , et al., Sci. Transl. Med. 2021, 13, eabc7804. 34516826 10.1126/scitranslmed.abc7804

[21] A. A. Walters , G. Santacana‐Font , J. Li , N. Routabi , Y. Qin , N. Claes , S. Bals , J. Tzu‐Wen Wang , K. T. Al‐Jamal , ACS Nano 2021, 15, 17549. 34677938 10.1021/acsnano.1c04456 PMC8613910

[22] K. J. Kauffman , J. R. Dorkin , J. H. Yang , M. W. Heartlein , F. DeRosa , F. F. Mir , O. S. Fenton , D. G. Anderson , Nano Lett. 2015, 15, 7300. 26469188 10.1021/acs.nanolett.5b02497

[23] T. Terada , J. A. Kulkarni , A. Huynh , S. Chen , R. van der Meel , Y. Y. C. Tam , P. R. Cullis , Langmuir 2021, 37, 1120. 33439022 10.1021/acs.langmuir.0c03039

[24] H. Zhang , X. Han , M. G. Alameh , S. J. Shepherd , M. S. Padilla , L. Xue , K. Butowska , D. Weissman , M. J. Mitchell , J. Biomed. Mater. Res. A 2022, 110, 1101. 35076171 10.1002/jbm.a.37356 PMC10155289

[25] O. Escalona‐Rayo , Y. Zeng , R. A. Knol , T. J. F. Kock , D. Aschmann , B. Slutter , A. Kros , Biomed. Pharmacother. 2023, 165, 115065. 37406506 10.1016/j.biopha.2023.115065

[26] M. M. T. Brusseler , A. Zam , V. M. Moreno‐Zafra , N. Rouatbi , O. W. M. Hassuneh , A. Marrocu , R. Liam‐Or , H. M. Abdel‐Bar , A. A. Walters , K. T. Al‐Jamal , Mol. Pharm. 2024, 21, 6339. 39556101 10.1021/acs.molpharmaceut.4c00875 PMC11615939

[27] E. M. Varypataki , K. van der Maaden , J. Bouwstra , F. Ossendorp , W. Jiskoot , AAPS J. 2015, 17, 216. 25387996 10.1208/s12248-014-9686-4 PMC4287297

[28] A. H. Rahman , D. K. Taylor , L. A. Turka , Immunol. Res. 2009, 45, 25. 19597998 10.1007/s12026-009-8113-x PMC4486050

[29] S. R. Mullins , J. P. Vasilakos , K. Deschler , I. Grigsby , P. Gillis , J. John , M. J. Elder , J. Swales , E. Timosenko , Z. Cooper , S. J. Dovedi , A. J. Leishman , N. Luheshi , J. Elvecrog , A. Tilahun , R. Goodwin , R. Herbst , M. A. Tomai , R. W. Wilkinson , J. Immunother. Cancer 2019, 7, 244. 31511088 10.1186/s40425-019-0724-8 PMC6739946

[30] J. S. Kurche , M. A. Burchill , P. J. Sanchez , C. Haluszczak , R. M. Kedl , J. Immunol. 2010, 185, 2106. 20639485 10.4049/jimmunol.1000172 PMC3863553

[31] J. S. Apostolico , V. A. S. Lunardelli , M. M. Yamamoto , E. Cunha‐Neto , S. B. Boscardin , D. S. Rosa , Front. Immunol. 2019, 10, 843. 31105693 10.3389/fimmu.2019.00843 PMC6492566

[32] J. Fucikova , D. Rozkova , H. Ulcova , V. Budinsky , K. Sochorova , K. Pokorna , J. Bartunkova , R. Spisek , J Transl Med 2011, 9, 223. 22208910 10.1186/1479-5876-9-223 PMC3259090

[33] R. M. Prins , H. Soto , V. Konkankit , S. K. Odesa , A. Eskin , W. H. Yong , S. F. Nelson , L. M. Liau , Clin. Cancer Res. Off. J. Am. Assoc. Cancer Res. 2011, 17, 1603. 10.1158/1078-0432.CCR-10-2563 PMC3071163 21135147

[34] Y. Nouri , R. Weinkove , R. Perret , J. Immunother. Cancer 2021, 9, 11e003065, 10.1136/jitc-2021-003065. PMC8606765 34799397

[35] G. Caron , D. Duluc , I. Fremaux , P. Jeannin , C. David , H. Gascan , Y. Delneste , J. Immunol. 2005, 175, 1551. 16034093 10.4049/jimmunol.175.3.1551

[36] A. E. Gelman , J. Zhang , Y. Choi , L. A. Turka , J. Immunol. 2004, 172, 6065. 15128790 10.4049/jimmunol.172.10.6065 PMC2833313

[37] M. Boes , F. Meyer‐Wentrup , Cancer Lett. 2015, 361, 49. 25697485 10.1016/j.canlet.2015.02.027

[38] Y. Qin , A. A. Walters , K. T. Al‐Jamal , Int. J. Pharm. 2023, 631, 122481. 36513254 10.1016/j.ijpharm.2022.122481

[39] J. De Waele , E. Marcq , J. R. Van Audenaerde , J. Van Loenhout , C. Deben , K. Zwaenepoel , E. Van de Kelft , D. Van der Planken , T. Menovsky , J. M. Van den Bergh , Y. Willemen , P. Pauwels , Z. N. Berneman , F. Lardon , M. Peeters , A. Wouters , E. L. Smits , Oncoimmunology 2018, 7, e1407899. 29399410 10.1080/2162402X.2017.1407899 PMC5790389

[40] L. Wen , B. Xin , P. Wu , C. H. Lin , C. Peng , G. Wang , J. Lee , L. F. Lu , G. S. Feng , Hepatology 2019, 69, 2518. 30693544 10.1002/hep.30528 PMC6541536

[41] T. Bald , J. Landsberg , D. Lopez‐Ramos , M. Renn , N. Glodde , P. Jansen , E. Gaffal , J. Steitz , R. Tolba , U. Kalinke , A. Limmer , G. Jonsson , M. Holzel , T. Tuting , Cancer Disc. 2014, 4, 674. 10.1158/2159-8290.CD-13-0458 24589924

[42] N. K. Brockwell , K. L. Owen , D. Zanker , A. Spurling , J. Rautela , H. M. Duivenvoorden , N. Baschuk , F. Caramia , S. Loi , P. K. Darcy , E. Lim , B. S. Parker , Cancer Immunol. Res. 2017, 5, 871. 28848054 10.1158/2326-6066.CIR-17-0150

[43] D. Tormo , A. Checinska , D. Alonso‐Curbelo , E. Perez‐Guijarro , E. Canon , E. Riveiro‐Falkenbach , T. G. Calvo , L. Larribere , D. Megias , F. Mulero , M. A. Piris , R. Dash , P. M. Barral , J. L. Rodriguez‐Peralto , P. Ortiz‐Romero , T. Tuting , P. B. Fisher , M. S. Soengas , Cancer Cell 2009, 16, 103. 19647221 10.1016/j.ccr.2009.07.004 PMC2851205

[44] H. Chen , D. L. Wang , Y. L. Liu , Mol. Med. Rep. 2016, 13, 2689. 26848042 10.3892/mmr.2016.4848

[45] S. Palchetti , D. Starace , P. De Cesaris , A. Filippini , E. Ziparo , A. Riccioli , J. Biol. Chem. 2015, 290, 5470. 25568326 10.1074/jbc.M114.601625 PMC4342463

[46] T. Inao , N. Harashima , H. Monma , S. Okano , M. Itakura , T. Tanaka , Y. Tajima , M. Harada , Breast Cancer Res. Treat. 2012, 134, 89. 22203435 10.1007/s10549-011-1930-3

[47] S. Y. Kwak , S. Lee , H. D. Han , S. Chang , K. P. Kim , H. J. Ahn , Mol. Pharm. 2019, 16, 4940. 31651174 10.1021/acs.molpharmaceut.9b00826

[48] Z. Luo , C. Wang , H. Yi , P. Li , H. Pan , L. Liu , L. Cai , Y. Ma , Biomaterials 2015, 38, 50. 25457983 10.1016/j.biomaterials.2014.10.050

[49] N. Rouatbi , A. A. Walters , P. M. Costa , Y. Qin , R. Liam‐Or , V. Grant , S. M. Pollard , J. T. Wang , K. T. Al‐Jamal , J. Control. Release 2024, 375, 776. 39284526 10.1016/j.jconrel.2024.09.019

[50] T. Nagato , Y. R. Lee , Y. Harabuchi , E. Celis , Clin. Cancer Res. Off. J. Am. Assoc. Cancer Res. 2014, 20, 1223. 10.1158/1078-0432.CCR-13-2781 PMC3956448 24389326

[51] A. Sica , A. Mantovani , J. Clin. Invest. 2012, 122, 787. 22378047 10.1172/JCI59643 PMC3287223

[52] R. L. Ball , K. A. Hajj , J. Vizelman , P. Bajaj , K. A. Whitehead , Nano Lett. 2018, 18, 3814. 29694050 10.1021/acs.nanolett.8b01101

[53] A. J. Geall , A. Verma , G. R. Otten , C. A. Shaw , A. Hekele , K. Banerjee , Y. Cu , C. W. Beard , L. A. Brito , T. Krucker , D. T. O'Hagan , M. Singh , P. W. Mason , N. M. Valiante , P. R. Dormitzer , S. W. Barnett , R. Rappuoli , J. B. Ulmer , C. W. Mandl , Proc. Natl. Acad. Sci. U S A 2012, 109, 14604. 22908294 10.1073/pnas.1209367109 PMC3437863

