# Supplementary material for: Triplet RNA Lipid Nanoparticles for Locoregional Cancer Immunotherapy
Source: Small Sci. 2025 Dec 12;6(1):e202500506. doi: 10.1002/smsc.202500506 (PMC12794677; doi:10.1002/smsc.202500506)
Supplement: Supplementary file 1 — Supplementary Material [file SMSC-6-e202500506-s001.pdf]

Supplementary Data

Triplet RNA Lipid Nanoparticles for Loco-regional Cancer Immunotherapy

Adam A Walters[1, *], Yue Qin[1], Amer F Saleh[2], Calvin C L Cheung[3], Qingyang Lyu[3] Ziyi Zhu[1], Hiba A M Gafar[1] Julie Tzu-Wen Wang[1], and Khuloud T Al-Jamal[1,3*]

1. Institute of Pharmaceutical Science, Faculty of Life Sciences & Medicine, King's College London, Franklin-Wilkins Building, 150 Stamford Street, London SE1 9NH, United Kingdom

2. Safety Sciences, Clinical Pharmacology and Safety Sciences, BioPharmaceuticals R&D, AstraZeneca, Cambridge, CB2 0AA, United Kingdom

3. JC STEM Lab of Nanomedicine for Advanced Therapy, 2/F, Laboratory Block, 21 Sassoon Road, Pokfulam, Hong Kong

[*]Corresponding author. Email: Khuloud.al-jamal@kcl.ac.uk; adam.walters@kcl.ac.uk

Amer F. Saleh is currently an employee of AstraZeneca and may or may not own stock options in the company.

Suppl. Table 1: Physical characteristics of pIpC-LNPs

	Size (d.nm)[a]	Surface Charge (mV)[a]	PDI[a]	Encapsulation Efficiency (%)[b]
pIpC-LNP	120.5±0.4	-7.17±4.8	0.08±0.03	78.1±3.8

a: size, polydispersity and charge of LNP were measured in 0.1X PBS with Zetasizer (Malvern Instruments).

b: Measured with the RiboGreen assay.

n=3 LNP per sample.

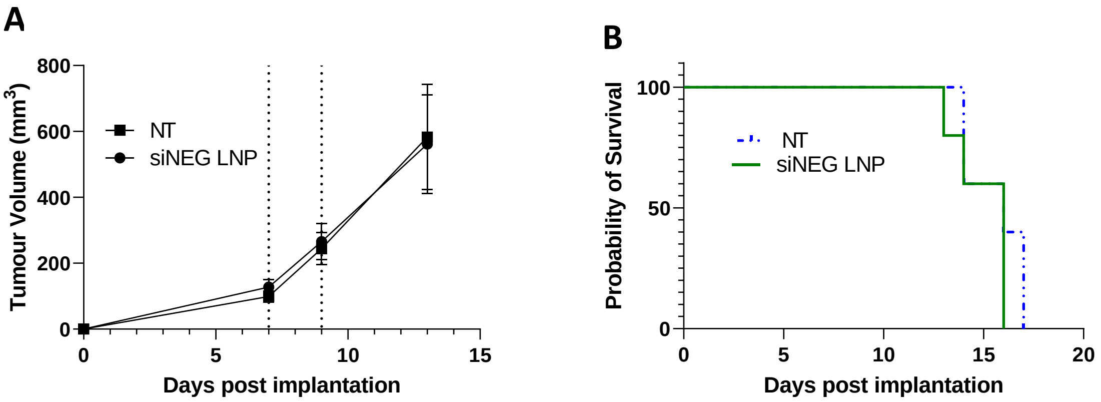

Figure S1: LNP administration intratumorally does not affect tumor growth. C57BL/6 mice (n=5) were implanted with B16F10 melanoma cells. At days 7 and 9 post implantation, mice were treated intratumorally with LNP incorporating siNEG (siNEG LNP, 15µg) or left untreated (NT). The mean±SEM tumor volume is plotted in each case **(A).** Survival of mice over the time course is plotted in the Kaplan-Meier graph **(B)**

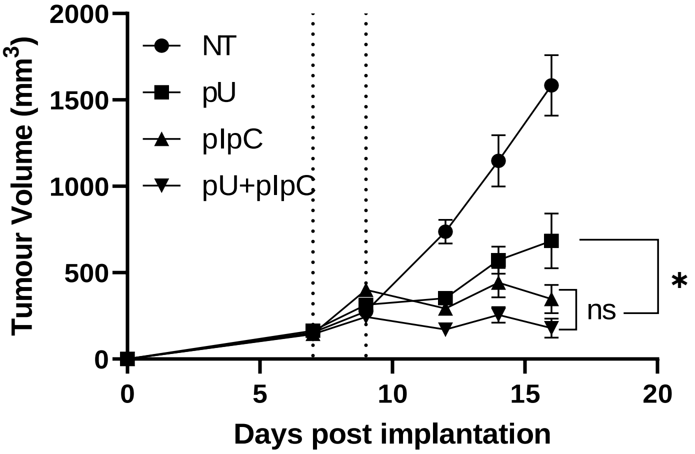

Figure S2: pIpC-LNP are more potent than TLR 7 agonist pU-LNP. C57BL/6 mice (n=7) were implanted with B16F10 melanoma cells. On days 7 and 9 post implantation were treated intratumorally with LNP incorporating either pIpC (15µg), pU (15µg), a combination of the two (pU+pIpC, 7.5µg+ 7.5µg) or left untreated (NT). Tumor growth was monitored with a digital caliper. Each point represents the mean±SEM of tumor volume. Statistical analysis was carried out using a two-way ANOVA, ns non-significant, *p<0.05.

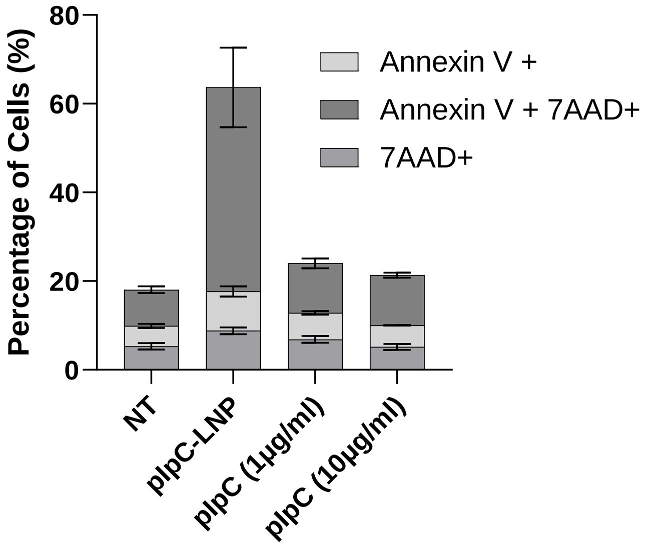

Figure S3: pIpC-LNP induces apoptosis in B16F10. B16F10 cells at 70-80% confluence were treated with lipid nanoparticle formulated pIpC (pIpC-LNP) (1µg/ml) or soluble pIpC (at 1µg/ml and 10 µg/ml). An additional group was left untreated (NT). Cells were harvested after 48 h of culture and stained with annexin V and 7AAD before being acquired on a FACs Celesta flow cytometer. Data analysis was performed using Flowjo software. The mean±SD percentage of cells staining positive for Annexin V and/or 7AAD is displayed.

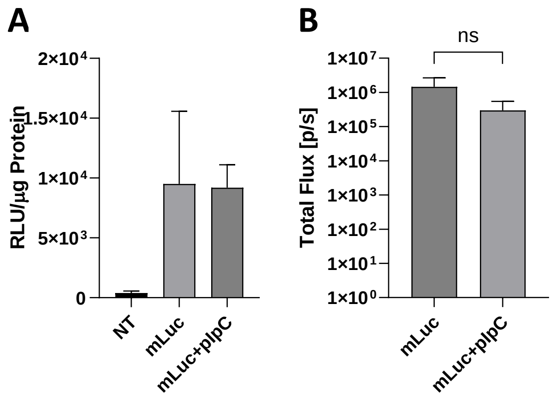

Figure S4: pIpC does not interfere with mRNA expression in B16F10. B16F10 cells were treated with LNP formulated with mLuc mRNA (1µg/ml) in the presence or absence of pIpC (1µg/ml). Following treatment (48 hours), cells were lysed via 3 freeze thraw cycles at -80°C. Cell extract was clarified by centrifugation and luciferase in supernatant was quantified by addition of D-Luciferin. Total protein in lysate was assessed by BCA assay with a standard curve of BSA. Both assays were read on a BMG LABTECH, FLUOstar® Omega plate reader. Data is expressed as relative light units per µg of extracted protein. Bars represent the mean + SD of three technical replicates (**A**). C57BL6 (n=2-3) were implanted with B16F10 subcutaneously, following 10 days tumor formation LNPs incorporating mLuc (15µg/mouse) were injected intratumorally in the presence or absence of LNP formulated with pIpC (15µg/mouse). Six hours following injection mice were injected subcutaneously with luciferin and imaged using an IVIS imaging system (Perkin Elmer). Mean and SD values obtained for total flux are presented in (**B**). Data was analyzed via Mann Whitney test ns=Non significant.
